# Population-based study of invasive Kingella kingae infections.

**DOI:** 10.3201/eid0601.000118

**Published:** 2000

**Authors:** P Yagupsky, R Dagan


into a 50-mL volume of broth reduces the concentration of inhibitory factors, facilitating isolation of the organism (4).

In 1993, we reported results of routine use of blood-culture bottles for processing cultures of exudates (7) at the Soroka University Medical Center, Beer-Sheva, Israel. From 1988 to 1992, 25 children with invasive *K. kingae* infections, defined as isolation of the organism from blood or normally sterile body fluids, were identified in southern Israel. From 1994 to 1998, 33 additional patients, including 32 children and a 21-year-old adult, were detected in the same area. Twenty-four (63.6%) of the 33 patients were male. Eight (24.2%) cases were diagnosed between January and June and 25 (75.8%) between July and December. Median age of children was 13 months (mean ± SD: 15.0 ± 7.6 months; range 6 to 37 months).

The fact that all children in southern Israel are born and receive inpatient medical services at the Soroka University Medical Center allowed us to calculate the incidence of invasive pediatric *K. kingae* infections in this population. During the 6-year period, the average annual number of births was 10,860. The annual incidence of invasive *K. kingae* infections during the same period was 11.9 per 100,000 in children $\leq$ 48 months of age, 19.2 per 100,000 in children <24 months of age, and 20.0 per 100,000 in infants <12 months of age.

When medical attention was sought, patients had been ill for a median of 3 days. Symptoms of upper respiratory tract infection were recorded in 12 (36.4%) children, stomatitis in 8 (24.2%), and diarrhea in 4 (12.1%). Occult bacteremia (positive blood culture with no obvious focal infection) was diagnosed in 16 children. In 15 children, *K. kingae* had invaded the bones. Septic arthritis was diagnosed in 11 children, involving the ankle in 4; the knee or wrist in 2 patients each; and the hip, shoulder, or elbow in one patient each. Osteomyelitis was diagnosed in two patients, affecting the femur in one and the tibia in the other. In two additional patients, both with fever and bacteremia, the location of the skeletal infection could not be determined. One limped and had tenderness over the femur, but X-rays and a $Technecium_{99}$-labeled bone scan showed no abnormalities. The other had pain in the heel but no fluid could be aspirated. Bacteremic tracheobronchitis occurred in one child, and endocarditis of the mitral valve was diagnosed in a 21-year-old woman who was receiving immunosuppressive therapy for systemic lupus erythematosus. All 33 patients were treated with ß-lactam drugs and recovered.

Injecting synovial fluid specimens into blood-culture bottles permitted the diagnosis of *K. kingae* in these patients and showed that this organism may be a common cause of invasive pediatric infections. The age distribution of the patients demonstrates that *K. kingae* is a pathogen of young children, especially those between the ages of 6 months and 2 years, among whom the incidence of invasive disease has remained stable since 1988. This age distribution of *K. kingae* infections parallels that for respiratory carriage of the organism. In a surveillance study among 48 children ages 6 to 42 months attending a day-care center in Israel, *K. kingae* was isolated from 109 (17.5%) of 624 throat cultures, and 34 children (70.8%) carried the organism at least once during an 11-month period (8). However, the organism was not detected in healthy infants ages 2 to 4 months attending a well-baby care clinic, which indicates some immunity to colonization and infection by *K. kingae* during the first months of life (8).

When the 1988 to 1993 surveillance data are added to those collected from 1994 to 1998, *K. kingae* infections show a significant seasonal pattern; 44 (75.9%) of 58 cases were diagnosed in the second half of the year ($p = 0.007$). This increase in *K. kingae* infections in winter has also been described in other respiratory pathogens. This finding, as well as the frequent detection of respiratory symptoms in children with invasive *K. kingae* infections, suggests that seasonal viral infections may facilitate the spread of *K. kingae* from the throat, to the bloodstream and bones. In a prospective study, *K. kingae* bacteremia was documented in 4 (13.7%) of 29 young children with culture-proven herpetic gingivostomatitis, confirming the role played by viral infections in the pathogenesis of infections caused by the organism (9).

With few exceptions, isolates of *K. kingae* remain susceptible to antibiotic drugs (10). Our results demonstrate that the prognosis of invasive *K. kingae* infections is generally good and patients respond promptly to appropriate antimicrobial therapy.

**Pablo Yagupsky and Ron Dagan**
Soroka Medical Center, Ben-Gurion University of the Negev, Beer-Sheva, Israel

### References

1. Graham DR, Band JD, Thornsberry C, Hollis DG, Weaver RE. Infections caused by *Moraxella*, *Moraxella urethralis, Moraxella*-like groups M-5 and M-6, and *Kingella kingae* in the United States, 1953-1980. Rev Infect Dis 1990;12:423-31.
2. deGroot R, Glover D, Clausen C, Smith AL, Wilson CB. Bone and joint infections caused by *Kingella kingae*: six cases and review of the literature. Rev Infect Dis 1988;10:998-1004.
3. Goutzmanis JJ, Gonis G, Gilbert GL. *Kingella kingae* infection in children: ten cases and review of the literature. Pediatr Infect Dis 1991;10:677-83.
4. Yagupsky P, Dagan R, Howard CB, Einhorn M, Kassis I, Simu A. High prevalence of *Kingella kingae* in joint fluid from children with septic arthritis revealed by the BACTEC blood culture system. J Clin Microbiol 1992;30:1278-81.
5. Birgisson H, Steingrimsson O, Gudnasson T. *Kingella kingae* infections in paediatric patients; five cases of septic arthritis, osteomyelitis and bacteraemia. Scand J Infect Dis 1997;29:495-8.
6. Lundy DW, Kehl DK. Increasing prevalence of *Kingella kingae* in osteoarticular infections in young children. J Pediatr Orthop 1998;18:262-7.
7. Yagupsky P, Dagan R, Howard CB, Einhorn M, Kassis I, Simu A. Clinical features and epidemiology of invasive *Kingella kingae* infections in southern Israel. Pediatrics 1993;92:800-4.
8. Yagupsky P, Dagan R, Prajgrod F, Merires M. Respiratory carriage of *Kingella kingae* among healthy children. Pediatr Infect Dis J 1995;14:673-8.
9. Amir J, Yagupsky P. Invasive *Kingella kingae* infection associated with stomatitis in children. Pediatr Infect Dis J 1998;17:757-8.
10. Jensen KT, Schonheyder H, Thomsen VF. In-vitro activity of ß-lactam and other antimicrobial agents against *Kingella kingae*. J Antimicrob Chemother 1994;33:635-40.

### Involving Ornithologists in the Surveillance of Vancomycin-Resistant Enterococci

**To the Editor:** Because migratory birds cross national or intercontinental borders, they are possible long-range vectors for human pathogens such as viruses, *Borrelia burgdorferi* sensu lato, and enteropathogenic bacteria with antibiotic resistance or virulence factors (1,2). Enterococci are ubiquitous in humans and animals and have a propensity for uptake and transfer of glycopeptide antibiotic resistance (3); therefore, the emergence of glycopeptide-resistant enterococci (GRE) in humans is a public health concern. Low-level vancomycin resistance (genotype *vanC-1-3*) is intrinsic in enterococcal species (e.g., *Enterococcus gallinarum, E. flavescens,* and *E. casseliflavus*) that may normally occur in the intestinal flora of some birds. However, the finding of high levels of GRE in wild birds suggests acquisition from an environmental source.

In March 1998, we obtained fecal samples while banding 318 northbound migrating gulls in Malmö, southern Sweden. Using a selective culture procedure with enrichment broth (bile esculin azide broth, Acumedia, LabFab, Ljusne, Sweden) containing vancomycin (8 µg/ml) and aztreonam (60 µg/ml), we isolated vancomycin-resistant *E. faecalis* from a black-headed gull (*Larus ridibundus*). High-level glycopeptide resistance (>256 µg/ml) was demonstrated by E-test (AB Biodisc, Solna, Sweden), and a *van*A genotype was found by polymerase chain reaction amplification (4). This survey protocol can also be used to detect medium to low levels of glycopeptide resistance. Using the same procedure in a study of 230 sub-Antarctic birds on Bird Island, South Georgia, in 1996, we found four GRE isolates with *vanC1* genotype (MIC 3-8 µg/ml).

Many species of gulls have moved into urban areas, where they commonly feed on human trash and deposit feces. The black-headed gull with GRE described above was banded as a fledgling in Malmö in 1995. Birds of this population spend the winter mainly in Western Europe (5), where they forage at garbage dumps, sewage outlets, and agricultural areas. This bird may have acquired GRE in such an area. *Van*A genotype *E. faecium* and *E. faecalis* have been found in poultry and pigs in the Netherlands and Denmark, where the vancomycin analog avoparcin has been used as a growth promoter (6). Manure from such farms may be a GRE source accessible to wild birds.

We have previously reported the introduction into Sweden of multidrug-resistant *Salmonella* Typhimurium by migratory birds (7). The present report further emphasizes the possibility of migratory birds as long-range vectors of bacteria potentially associated with human disease. The risk to humans for GRE from migratory birds may seem insignificant compared with such risk from hospitalization or from eating meat products from GRE-colonized
